# Identification and characterization of ERV-W-like sequences in Platyrrhini species provides new insights into the evolutionary history of ERV-W in primates

**DOI:** 10.1186/s13100-020-0203-2

**Published:** 2020-02-01

**Authors:** Nicole Grandi, Maria Paola Pisano, Martina Demurtas, Jonas Blomberg, Gkikas Magiorkinis, Jens Mayer, Enzo Tramontano

**Affiliations:** 1 Department of Life and Environmental Sciences, University of Cagliari, Cittadella Universitaria di Monserrato, SS554, Monserrato, Cagliari Italy; 2 Department of Medical Sciences, Uppsala University, Uppsala, Sweden; 3 Institute of Human Genetics, University of Saarland, Homburg, Germany; 4 Department of Hygiene, Epidemiology, and Medical Statistics, Medical School, National and Kapodistrian University of Athens, Athens, Greece; 5 Istituto di Ricerca Genetica e Biomedica (IRGB), CNR, Monserrato, Cagliari Italy

**Keywords:** ERV-W, Platyrrhini, Catarrhini, Primate evolution, Endogenous retrovirus, HERV phylogeny, ERV1–1, Pre-gag, 5′ leader

## Abstract

**Background:**

Endogenous Retroviruses (ERVs) constitute approximately 8% of every human genome and are relics of ancestral infections that affected the germ line cells. The ERV-W group contributed to primate physiology by providing an envelope protein (Syncytin-1) that has been adopted for placenta development in hominoids. Expression of Human ERV-W (HERV-W) sequences is investigated for a pathological role in various human diseases.

**Results:**

We previously characterized ERV-W group genomic sequences in human and non-human Catarrhini species. We now investigated ERV-W-like sequences in the parvorder Platyrrhini, especially regarding two species with complete genome assemblies, namely marmoset (*Callithrix jacchus*) and squirrel monkey (*Saimiri boliviensis*). We identified in both species proviral sequences, annotated as ERV1–1 in respective genome assemblies, sharing high sequence similarities with Catarrhini ERV-W. A total of 130 relatively intact proviruses from the genomes of marmoset and squirrel monkey were characterized regarding their structural and evolutionarily relationships with Catarrhini ERV-W elements. Platyrrhini ERV-W sequences share several structural features with Catarrhini ERV-W elements and are closely related phylogenetically with the latter as well as with other ERV-W-related gammaretrovirus-like ERVs. The ERV-W group colonized Platyrrhini primates of both Callitrichidae and Atelidae lineages, with provirus formations having occurred mostly between 25 and 15 mya. Two LTR subgroups were associated with monophyletic proviral bodies. A *pre-gag* region appears to be a sequence feature common to the ERV-W group: it harbors a putative intron sequence that is missing in some ERV-W loci, holding a putative ORF as well. The presence of a long *pre-gag* portion was confirmed among all gammaretroviral ERV analyzed, suggesting a role in the latter biology. It is noteworthy that, contrary to Catarrhini ERV-W, there was no evidence of L1-mediated mobilization for Platyrrhini ERV-W sequences.

**Conclusions:**

Our data establish that ERV-W is not exclusive to Catarrhini primates but colonized both parvorders of Simiiformes, providing further insight into the evolution of ERV-W and the colonization of primate genomes.

## Background

Endogenous Retroviruses (ERVs) are integrated DNA relics from retroviral infections that affected mammalian ancestors for at least 100 million years (my) [1, 2]. Such infections have been caused by exogenous retroviruses - now mostly gone extinct – that targeted germ line cells, resulting in stably inherited proviruses in those host genomes. ERVs have been inherited in a mendelian fashion throughout the offspring, and sequences derived from human ERVs (HERVs) constitute about 8% of our genomic DNA [3]. Similar to exogenous retroviruses, ERVs are usually comprised of *gag*, *pro*, *pol* and *env* genes flanked by two Long Terminal Repeats (LTRs) and formed during reverse transcription of retroviral RNA into a double-stranded DNA.

Among the various HERVs (see for example [4] for an updated classification) the HERV-W group gained considerable attention especially because of a provirus in human chromosome 7q21.2 (named ERVW-1) encoding a functional Env protein that has been coopted during evolution for placenta development and homeostasis [5, 6]. The HERV-W group has been also intensively investigated for links to human diseases, with a special emphasis on cancer and autoimmune/inflammatory disorders [1, 7–10]. HERV-W may be involved in multiple sclerosis (MS) given that the Env surface subunit was shown to have pro-inflammatory effects that might contribute to damage of various brain cell populations (recently reviewed in [7]). Accordingly, HERV-W Env overexpression led to development of allergic encephalomyelitis in mice [11], while treatment with a monoclonal antibody against HERV-W Env rescued myelin expression [12], a phenomenon currently investigated as an innovative clinical approach for treating MS [13].

Overall, however, the pathological role of HERV-W as well as of the other HERV groups is uncertain, still lacking definitive associations between specific retroviral elements and human disorders. In principle, one of the possible problems in assessing the physio-pathological significance of HERV groups’ expression is the frequent poor knowledge about the position and nucleotide sequence of their individual members, preventing the specific assessment of each HERV locus transcriptional activity. In the light of the possible relevance of the HERV-W group in human pathogenesis, we had characterized in more detail HERV-W loci present in human genome assembly GRCh37/hg19 [14]. To better depict the spread of ERV-W in primates, we had also investigated ERV-W sequences in non-human Catarrhini species (Additional file 1: Figure S1) [15]. The latter lineage includes hominoids and old world monkeys, and is estimated to have diverged from Platyrrhini approximately 40 million years ago (mya) [16, 17] (Additional file 1: Figure S1).

Our previous analysis showed that the ERV-W group spread within the entire Catarrhini parvorder, with a high proportion of ERV-W elements being orthologues of the 213 human loci investigated as well as numerous species-specific insertions lacking an orthologous locus in humans [15]. As for the Platyrrhini parvorder, previous studies concluded that ERV-W colonized Catarrhini primates after their evolutionary separation from Platyrrhini, seemingly supported by a lack of ERV-W sequences in Platyrrhini species as well as *Prosimians* [18, 19]. A single study had reported presence of ERV-W LTRs (but not internal portion) in New World Monkeys [20].

We re-examined presence of ERV-W sequences in Platyrrhini species by analyzing assembled genome sequences of marmoset (*Callithrix jacchus*) and squirrel monkey (*Saimiri boliviensis*) (Additional file 1: Figure S1).

We characterized sequences of identified ERV-W like elements by analysis of proviral structures of respective ERV-W loci, consensus sequences, estimates of proviral ages, phylogenetic analysis. We established close relationship of Platyrrhini ERV-W sequences with Catarrhini ERV-W as well as other closely related ERV groups. We furthermore investigated presence and evolutionary origins of a sequence region between the 5’LTR and the *gag* gene, named pre-*gag*, that may represent a functionally relevant sequence feature shared by several gammaretroviruses. Our analysis demonstrates spread of endogenous retroviral sequences very similar in sequence to Catarrhini ERV-W also in the Platyrrhini lineage and provides further insight into the evolution of ERV-W during those colonizations.

## Results

### Collection of ERV-W-like proviral sequences from marmoset and squirrel monkey genome sequence assemblies

As detailed in materials and methods, ERV-W-like sequences present in marmoset and squirrel monkey genome assemblies – named ERV1–1 according to RepBase – were previously retrieved from UCSC Genome Browser [21] by BLAT searches [22] using HERV-W group reference sequences (HERV17 and LTR17) obtained from RepBase Update [23] as a query [15]. To the best of our knowledge, ERV1–1 sequences were so far not investigated in the HERV-W context. Besides the sequences annotated as ERV1–1 in marmoset reference genome, other LTR retrotransposons designated as “ERV1–1” in RepBase Update [23] corresponded to ERV groups from at least 28 vertebrate species, which included another primate species, namely *Tarsius syrichta*. A Blat search in each of those vertebrate genome assemblies using the proviral consensus previously built from marmoset and squirrel monkey datasets as a query [15] did not establish significant sequence similarities, corroborating that ERV-W is limited to certain primate lineages, as described before [14, 15], and furthermore appears to be present also in marmoset and squirrel monkey, with respective sequences annotated as ERV1–1 elements (data not shown). For this reason, we will refer to these elements in marmoset and squirrel monkey as ERV-W-like sequences, also in order to avoid confusion in the light of not directly related other ERV1–1 sequences in other vertebrates.

### Structural characterization of ERV-W-like proviral sequences

In order to build a dataset that includes the most intact ERV-W-like proviruses, retrieved sequences were analyzed by dot-plot comparisons with the ERV1–1 group RepBase reference sequence. A total of 130 proviruses (59 from marmoset, 71 from squirrel monkey) harboring reasonably intact LTRs and internal portions were selected for subsequent analysis (Additional file 5). Our initial analysis also addressed ERV-W-like sequences with shortened LTRs. Partially truncated LTRs can be a hallmark of sequences that are actually processed pseudogenes formed by LINE-1-mediated retrotransposition of ERV proviral transcripts. While HERV-W processed pseudogenes were abundantly formed during Catarrhini primate evolution [14, 15, 24], in contrast, Platyrrhini ERV-W-like elements with shorter LTRs did not display specific hallmarks of processed pseudogenes, i.e. no deletion of the U3 region for 5’LTRs and no deletion of the U5 region for 3’LTRs, as well as no poly-A tail downstream from the 3’LTR [24] (data not shown). This suggests that, contrary to ERV-W proviral transcripts in Catarrhini, LINE-1 machinery did not retrotranspose Platyrrhini ERV-W proviral transcripts. Besides proviral LTRs, we also estimated solitary LTR abundance in marmoset and squirrel monkey genome assemblies and identified a total of 176 and 164 solitary LTRs, respectively (data not shown). Hence, considering 59 ERV-W-like proviral loci in marmoset and 71 in squirrel monkey genomes solitary LTRs appear as 3 and 2.3 times, respectively, more frequent than proviral sequences in those genomes.

Then, to gain further insight into sequence similarities of Platyrrhini ERV-W-like sequences and Catarrhini ERV-W elements, we examined provirus structures of the above mentioned 130 Platyrrhini ERV-W-like sequences, also considering that, to the best of our knowledge, no detailed information about this Platyrrhini ERV group is currently available in the published literature.

**Fig. 1:**
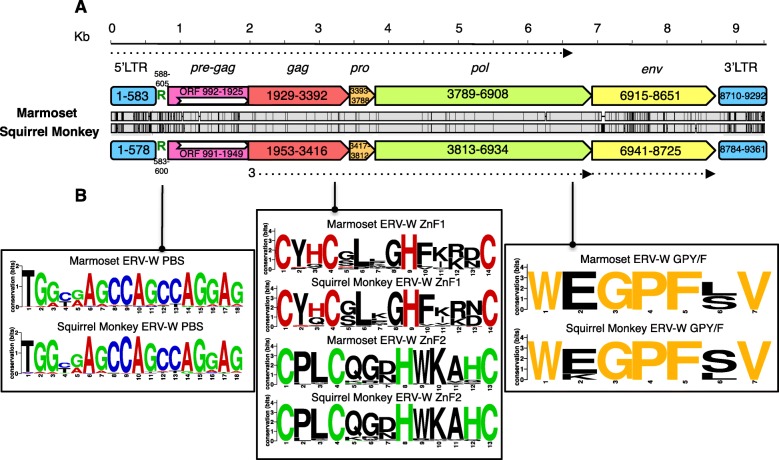
*Structural features of Platyrrhini ERV-W proviruses.* In panel A, presence and nucleotide positions of selected ERV-W structural elements are depicted for proviral consensus sequences generated from marmoset (CalJac) and squirrel monkey (SaiBol) ERV-W datasets. Nucleotide differences between both consensuses are indicated as vertical lines between the two provirus maps. Coordinates of a putative ORF identified within the *pre-gag* region are also annotated. The reading frame for translation of Gag, Pro and Pol proteins is indicated by a dotted arrow. Typical for retroviruses, Env is very likely translated from a spliced *env* mRNA. Panel B depicts selected sequence features in marmoset and squirrel monkey ERV-W sequences: a PBS predicted to be specific for tRNA^Arg^ (found in 81% and 84,5% of ERV-W elements, respectively); Gag nucleocapsid zinc fingers I (found in 63 and 33% of ERV-W elements, respectively) and II (found in 51 and 52% of ERV-W elements, respectively); and Pol integrase GPY/F (found in 42 and 35%, respectively). Respective motifs were counted as present only in the absence of internal stop codons and amino acid substitutions at the specific residues

Main retroviral features and coding regions of marmoset and squirrel monkey ERV-W-like elements were further characterized in respective consensus sequences generated previously, both approximately 9.3 kb in length [15] (Fig. 1, panel A). Briefly, both consensus sequences showed a classical proviral structure, in which *gag*, *pro*, *pol* and *env* genes, all located in the same reading frame, are flanked by 5′ and 3’LTRs with a length of about 600 nucleotides. The PBS sequence, binding a specific tRNA that primes the reverse transcription process [25], is 18 nucleotides long and was predicted to recognize an arginine (Arg; R) tRNA (Fig. 1, panel A). The same tRNA type was already predicted to be the second most frequent one for HERV-W elements after the canonical PBS for tryptophan (W) tRNA [14]. We further analyzed PBS sequences in individual marmoset and squirrel monkey ERV-W-like proviral sequences. The PBS region was present in 81 and 85% of ERV-W loci in marmoset and squirrel monkey, respectively (Fig. 1, panel B), and was confirmed to share the highest similarity with tRNA-Arg in all cases except one locus in each species, displaying had a PBS with highest similarity to Lysine tRNA. Although the canonical PBS type should be W one, it must also be noted that the PBS sequence for tRNA^Arg^ is just slightly different from that of tRNA^Trp^, and sometimes the two codons may even overlap due to a single nucleotide shift [4].

Besides the common proviral genes, both consensus sequences showed an atypical *pre-gag* portion between the 5’LTR-PBS region and the *gag* gene. The *pre*-*gag* portion was previously identified as a common structural feature of almost all (H)ERV-W elements [14, 15] (Fig. 1, panel A). The *pre-gag* of Catarrhini ERV-W sequences harbored a putative ORF starting in the *pre-gag* portion and extending into the *gag* and *pro* genes (nucleotides 1927 to 4305 in the HERV-W proviral consensus) [14, 15]. RetroTector software [26] predicted a putative ORF also within the Platyrrhini ERV-W *pre-gag*, ranging from nt 992–1925 and nt 991–1949 of marmoset and squirrel monkey consensus sequences, respectively (Fig. 1). A similar putative ORF, ranging from nt 926–1838, was also inferred from the RepBase reference sequence (ERV1–1_CJa). The conserved presence of the *pre-gag* region in both Platyrrhini and Catarrhini ERV-W elements and the possible presence of a coding ORF prompted us to further investigate the *pre-gag* sequence in relation to other gammaretrovirus-like ERVs (see further below).

Presence of other taxonomically significant structural features known to be shared by all class I gammaretroviruses [27] was also investigated (Fig. 1, panel B). Briefly, typical gammaretroviral features include one or two Gag NC zinc fingers, involved in the packaging of the retroviral RNA genome [28], and a Pol IN C-terminal GPY/F motif, which binds the host DNA during provirus integration [29]. In addition, a biased nucleotide composition is often found, possibly due to cellular editing systems acting on the encapsidated viral RNA [10, 27, 30]. Our analysis revealed the presence of the following structural features in the two proviral consensus sequences representing marmoset and squirrel monkey ERV-W-like elements: i) one Gag NC zinc finger with a Cx_2_Cx_4_Hx_4_C amino acid sequence at nt 3219–3260 and nt 3243–3284, respectively; ii) a second modified Gag NC zinc finger characterized by loss of one of the variable residues (Cx_2_Cx_3_Hx_4_C) (as previously reported for both HERV-H [31] and HERV-W [14] groups) at nt 3291–3329 and nt 3315–3353, respectively; iii) a C-terminal Pol IN GPY/F motif with a canonical WxGPFxV amino acid composition, at nt 6685–6705 and nt 6711–6731, respectively (Fig. 1, panel B). Presence of above features was assessed for each marmoset and squirrel monkey ERV-W proviral sequence harboring respective proviral regions, disregarding motifs with internal stop codons and substitutions of specific residues. Gag zinc finger I was present in 63 and 33% of marmoset and squirrel monkey ERV-W elements, respectively, while Gag zinc finger II was found in 51 and 52% of elements, respectively. Of note, besides the preserved specific residues, Gag zinc finger II showed a higher degree of sequence conservation at the motif’s variable residues, which were less conserved for Gag zinc finger I (Fig. 1, panel B). A GPY/F motif within the Pol IN was identified in 42 and 35% of marmoset and squirrel monkey ERV-W proviruses, respectively, showing almost equal conservation at the various aa positions (Fig. 1, panel B). Finally, as for nucleotide composition of ERV-W-like sequences, we detected a mild nucleotide bias towards purines, with an enrichment of A nucleotides (~ 28%) and a depletion of G nucleotides (~ 22%), as already reported for Catarrhini ERV-W [14] and HERV-K [32] groups (data not shown). The frequency of pyrimidines was instead around 25% for both T and C bases (data not shown).

### LTR phylogeny and subgroup classification

Platyrrhini ERV-W-like elements retrieved from marmoset and squirrel monkey genome sequences display high nucleotide similarities with internal portions of Catarrhini ERV-W, yet are more diverged for LTR sequences [15]. As previously observed for the HERV-W group, as well as for HERV-K HML-1 and HML-5 elements, such differences might be due to independent and faster evolution of LTR sequences during endogenization periods generating different LTR subtypes associated with monophyletic proviral bodies [15, 33, 34]. We analyzed the phylogeny of Platyrrhini ERV-W-like proviral LTRs as well as *gag*, *pol* and *env* genes by neighbor joining (NJ) analysis. As already observed for Catarrhini ERV-W sequences [14], phylogenetic analysis of retroviral genes did not identify statistically well supported clusters, thus suggesting monophyly (data not shown). However, NJ trees of 5′ and 3′ LTR sequences identified at least 2 major, phylogenetically distinct LTR (sub)groups (named here A and B) thus supporting the existence of different LTR subtypes associated with monophyletic proviral bodies (Additional file 2: Figure S2). Particularly, LTRs belonging to subgroups A and B were well supported by bootstrap values of 99 and 90, respectively. Subgroup B could moreover be divided into two clusters, named B1 and B2, with 99 and 68% bootstrap support, respectively. Aside from these subgroups, the remaining LTRs grouped into smaller clusters with proviral 5′ and 3′ LTRs often grouped very close to each other and with relatively high bootstrap values (Additional file 2: Figure S2).

**Fig. 2:**
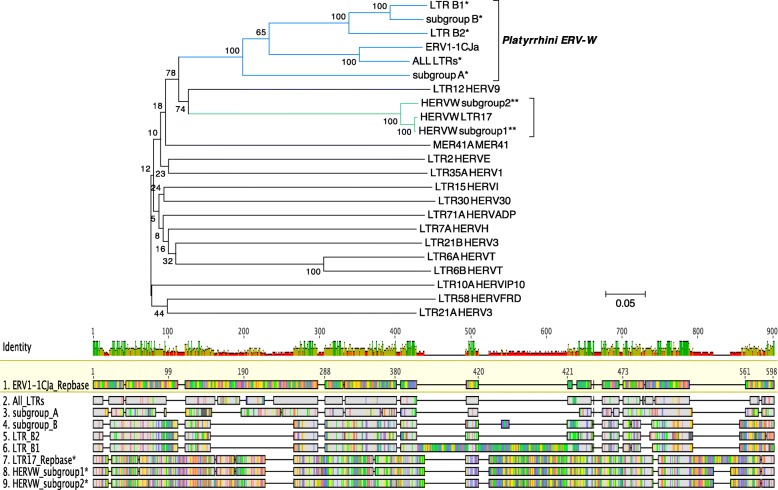
*Phylogenetic analysis of LTR consensus sequences.* Nucleotide consensus sequences generated for each Platyrrhini ERV-W LTR subgroup (“*”) were analyzed employing the NJ method and the Kimura-2-parameter model applying pairwise deletion. The LTR subgroup consensus generated for Catarrhini ERV-W (“**”) [14] and RepBase reference sequences for other class I ERV groups were also included. Phylogeny was tested employing the bootstrap method with 1000 replicates. The length of branches indicates numbers of substitutions per site. A multiple sequence alignment of consensus sequences of Platyrrhini ERV-W LTR subgroups and the RepBase reference sequence is depicted below the tree, with nucleotide substitutions represented by coloured vertical lines

In order to characterize sequence differences of above defined Platyrrhini ERV-W LTR subgroups, we generated a general LTR consensus (from the alignment of all LTRs included in the NJ tree, see Additional file 2: Figure S2) as well as A and B subgroup-specific consensus sequences that were compared to the RepBase reference (ERV1–1_CJa-LTR) through a multiple alignment and an NJ phylogenetic tree (Fig. 2). Consensus sequences of the two HERV-W subgroups, as reported previously [14], and RepBase references for the other class I HERV groups were also included in the analysis (Fig. 2). The Platyrrhini ERV-W-like LTR reference from RepBase (ERV1–1 CJa) and the general and subgroup-specific consensuses, as generated from our sequence datasets, clustered together with a 100% bootstrap support (Fig. 2). Within this cluster, the general LTR consensus (as built from the overall squirrel monkey and marmoset proviral dataset following majority rule) is most related to the RepBase reference (100% bootstrap support) (Fig. 2). Subgroup A LTR consensus appeared instead to be more diverged with respect to the RepBase reference, while subgroup B LTR as well as both B1 and B2 consensus sequences share a deletion of about 110 nt, corresponding to nt 146–255 of the RepBase reference sequence. B1 elements are further characterized by a ~ 200 nt insertion found in approximately 80% of B1 elements. Analysis of such insertion using CENSOR [35] revealed sequence similarities with different repetitive elements, among them Gypsy and HERVIP10. The presence or absence of the ~ 200 nt insertion was also responsible for definition of two separate branches within the B1 cluster in the NJ tree (Additional file 2: Figure S2). Finally, our NJ analysis further confirmed the relatively low level of sequence identities between LTR sequences associated with Catarrhini and Platyrrhini ERV-W internal portions (Fig. 2).

### Estimating time of integration

**Fig. 3:**
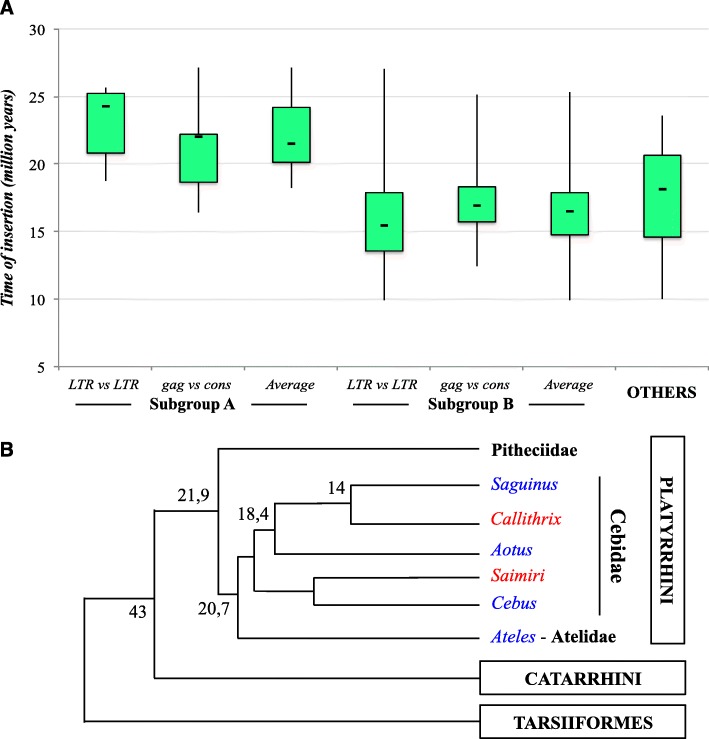
*Time of integration of ERV-W sequences in* Platyrrhini *primates.* In panel A, time periods of colonization for each Platyrrhini ERV-W subgroup as well as the sequences not clustering in any supported subgroup (“others”) were estimated through nucleotide divergence using a neutral substitution rate (see Material and Methods). In panel B, Platyrrhini genera including species analyzed in this manuscript are indicated in red, while other Platyrrhini genera with evidence of ERV-W sequences but lacking genome reference assemblies are indicated in blue. Phylogenetic relationships and estimated time periods of separation of evolutionary lineages are indicated (in millions of years ago, as derived from [16, 17]

The time of integration of marmoset and squirrel monkey ERV-W proviruses whose LTRs clustered in the above-mentioned subgroups (*n* = 46) was estimated by two different approaches based on a molecular clock, one based on LTR-LTR sequence divergence and another one based on sequence divergence to a *gag* gene consensus specific for each subgroup, as detailed in materials and methods. Hence, the ERV-W proviruses that were not included in any subgroup based on phylogenetic analyses were evaluated employing only LTR-LTR sequence divergence, due to the low reliability of a consensus built from a heterogeneous ensemble of sequences. With no well-established nucleotide substitution rate (SR) for Platyrrhini available, we estimated ages based on the human neutral SR (0.45% substitutions/nucleotide/million year), which has been previously used to estimate ages of ERVs in primates [36]. Results indicated that Platyrrhini marmoset and squirrel monkey genomes were colonized by ERV-W like sequences roughly between 25 and 15 mya (Fig. 3, panel A), with an averaged age of 18 my. Subgroup A members seemed to represent the first wave of insertions, being significantly older than the other ERV-W like loci based on a Student’s *t*-test (*p* = 0.000018). With overlapping time periods of integrations in mind, we searched for homologous ERV-W-like loci shared between marmoset and squirrel monkey genome sequences. We identified at least 19 orthologous ERV-W-like insertions (data not shown), confirming that a proportion of ERV-W-like loci has been acquired before the evolutionary separation of the two Platyrrhini lineages that is thought to have occurred between 20 and 18 mya [16, 17].

As estimates of ages based on a molecular clock provide relatively rough numbers on ERV colonization of genomes, we complemented our analysis by searching for similar sequences in species closely related to marmoset and squirrel monkey. We performed BLASTn searches (discontiguous megablast) in the NCBI nucleotide collection (nr/nt) for Platyrrhini species other than marmoset and squirrel monkey, specifically nucleotide sequences derived from families Atelidae, Cebidae and Pitheciidae, using the RepBase CalJac reference sequence as a query (data not shown). Preliminary evidence of ERV-W-like sequences was found for Platyrrhini species belonging to Cebidae (*Aotus lemurinus*, *Aotus nancymaae*, *Aotus trivirgatus*, *Cebus capucinus imitator*, *Saguinus midas*) and Atelidae (*Ateles belzebuth*), but not in Pitheciidae species (as well as in Tarsiiformes) (Fig. 3, panel B). These results further support that ERV-W-like elements colonized respective primate genomes between 25 and 15 mya.

### Phylogenetic relationships between Platyrrhini ERV-W and other gammaretroviral ERVs

Considerable sequence identity between ERV-W sequences in Catarrhini primates and sequences identified in marmoset and squirrel monkey strongly suggested closer evolutionary relationships between those ERVs. Phylogenetic analysis of Gag, Pol and Env putative proteins (puteins) obtained by RetroTector analysis [4, 37] of respective consensus sequences corroborated these relationships, as previously reported [15]. We further evaluated such close phylogenetic relationship now focusing on the putative Reverse Transcriptase - Ribonuclease H (RT-RH) amino acid sequence, which is known to be one of the most conserved among Retroviridae species. Marmoset and squirrel monkey proviral consensuses [15] as well as the Catarrhini HERV-W proviral consensus generated from the human dataset [15] were used to infer and translate the RT-RH amino acid sequence (see materials and methods for details). Other gammaretroviral-like HERV RT-RH portions were extracted from Pol consensus amino acid sequences reconstructed for each HERV group by RetroTector and based on the most intact insertions present in human genome assembly GRCh37/hg19 [4]. All the resulting RT-RH amino acid sequences were multiply aligned and further analyzed by constructing a maximum-likelihood (ML) phylogenetic tree (Additional file 3: Figure S3). The overall tree topology confirmed that RT-RH amino acid sequences inferred for marmoset and squirrel monkey ERV-W sequences are closely related phylogenetically with HERV-W RT-RH, and are furthermore closely related, with maximum bootstrap values, to other ERV groups with an already established phylogenetic relationship to HERV-W, specifically HERV-9 and HERV-30 (Additional file 3: Figure S3, blue branches).

### Characterization of the ERV-W pre-gag region

**Fig. 4:**
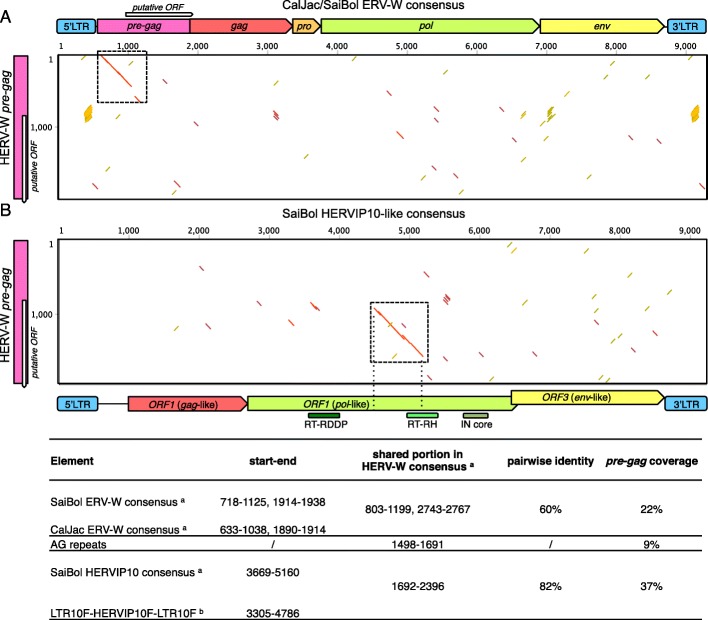
*Composition of ERV-W pre-gag nucleotide sequence.* The Catarrhini ERV-W *pre-gag* sequence was compared to Platyrrhini ERV-W consensus sequence **a** and to HERVIP10 **b** by dotplots. The putative ORF sequence within the *pre-gag* of Platyrrhini and Catarrhini ERV-W is indicated. Note the different location of that sequence within *pre-gag*. Regions within ERV-W *pre-gag* informative regarding the evolution of *pre-gag* are boxed. Nucleotide portions shared between sequences are indicated by red dots, lines and further detailed in the table below. ^a^ consensus sequences generated from the proviral datasets used in this study; ^b^ reference sequences retrieved from RepBase

As described here and previously [14, 15], ERV-W sequences in human and non-human Catarrhini primates are characterized by an approximately 2 kb long *pre-gag* region located between PBS and *gag* gene. A portion of that *pre-gag* region was also found in marmoset and squirrel monkey ERV-W proviruses [15]. We now further analyzed the *pre-gag* region *in* Catarrhini and Platyrrhini. Notably, more pronounced sequence similarities were limited to approximately 400 nt at the 5′ end when compared to HERV-W *pre-gag* (Fig. 4). This also means that the putative ORF inferred for Platyrrhini ERV-W *pre-gag*, located for the most part in the downstream *pre-gag* region, is different in sequence compared to the one predicted for Catarrhini primates (Fig. 4).

In order to gain further insight into the origin of the remaining approximately 1.5 kb of the Catarrhini ERV-W *pre-gag* region that is different in sequence from the Platyrrhini ERV-W *pre-gag*, we performed a BLAT search with such ~ 1.5 kb probe sequence in marmoset and squirrel monkey genome assemblies. A ~ 650 nt long region of the Catarrhini ERV-W *pre-gag* displayed highly scoring matches with another ERV group, identified as HERVIP10 by RepeatMasker analysis (data not shown). More specifically, the particular *pre-gag* portion displayed 82% sequence identity with a central portion of HERVIP10F ORF2 (nt 2786–4249 in the RepBase HERVIP10F reference sequence) (Fig. 4). The HERVIP10F ORF2 encodes for a Pol-like protein, and the sequence shared with Catarrhini ERV-W *pre-gag* corresponds to the 5′ portion of the RH domain based on results obtained from RetroTector analysis and NCBI Conserved Domain Search tool [38] (Fig. 4). Catarrhini and Platyrrhini ERV-W thus share a *pre-gag* region of approximately 400 nt, while the Catarrhini ERV-W *pre-gag* harbors an additional region that is missing in Platyrrhini ERV-W*.* That additional region appears to derive from the (former) *pol* gene region of an HERVIP10-like ERV group present in Platyrrhini*.* It is reasonable to speculate that the latter portion was acquired through a recombination event that occurred after the separation from Catarrhini. However, we note that an ERV-W locus on the chimpanzee Y chromosome, nt 21,951,590-21,956,101 (assembly Feb. 2011 - CSAC 2.1.4/panTro4), harbors a *pre-gag* sequence that has further 350 shared nucleotides in addition to the above 400, and lacks the downstream AG-rich repeat and the HERVIP10-like portion, thus being more similar to Platyrrhini ERV-W *pre-gag* sequence than to the one normally found in Catarrhini. In addition, the LTRs of that element (annotated as LTR12F) showed relatively high nucleotide similarity (55% versus the overall 34% observed with “canonical” HERV-W LTRs) with Platyrrhini ERV-W LTRs. Comparative genomic analysis localized the sequence orthologous to this locus in human chromosome Yq11.221, nt 14,340,494-14,345,004 (assembly GRCh38/hg38), likewise annotated as LTR12F-HERV17-LTR12F. That human locus and other elements with similar structure were previously included in a sequence dataset of Catarrhini ERV-W elements showing low score identity to HERV17 [15], being more similar to Platyrrhini ERV-W sequences.

**Fig. 5:**
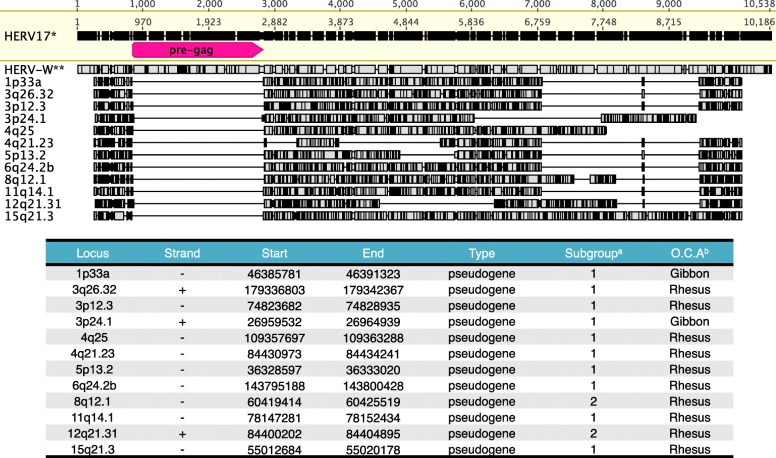
Catarrhini *ERV-W sequences lacking the pre-gag portion.* Multiple sequence alignment, chromosomal bands and genomic coordinates (as referred to GRCh38/hg38 genome assembly) of ERV-W processed pseudogenes in which the *pre-gag* sequence is absent entirely. Sequence differences compared to the HERV17 sequence are indicated. Numbers of rulers refer to nt positions for HERV17 and the multiple alignment. The *pre-gag* region is indicated for the HERV17 sequence. ^a^ based on a classification reported in [14]. ^b^ Most basal species with orthologous locus

Finally, it is interesting to note that a minority of HERV-W loci, all of them representing processed pseudogenes, lacks the *pre-gag* region entirely (Fig. 5). Absence of *pre-gag* was also confirmed for the corresponding non-human Catarrhini primate orthologous loci (data not shown). Because of the fact that all the (H)ERV-W loci lacking the *pre-gag* portion are actually processed pseudogenes we hypothesized that the *pre-gag* portion has been removed occasionally through the splicing of proviral transcripts originating from one or several source elements. Thus the *pre-gag* region may represent an intron sequence. Accordingly, the *pre-gag* region being an intron is supported by remarkable sequence similarities with splice donor (SD) and splice acceptor (SA) sites (Additional file 4: Figure S4, panel A). The missing *pre-gag* region coincides with 5’GT…AG3’ boundaries typically seen for intron 5′ and 3′ ends, respectively (Additional file 4: Figure S4, panel A). Sequences upstream and downstream of actual splice sites are also largely in accord with sequence conservation around splice sites, as found for both the RepBase HERV17 reference sequence and for a consensus sequence generated from the complete dataset of 65 HERV-W proviruses present in the human genome (Additional file 4: Figure S4, panel B). Furthermore, when considering those proviruses harboring the *pre-gag* region, the typical nucleotide composition of splice sites is conserved in a large majority of sequences, specifically SD (5’GT3’, 42/44 proviruses), branch site (5’CTA/GAC/T3’, 42/48), and SA (5’AG3’, 42/44) (Additional file 4: Figure S4, panel B). Thus, as the great majority of HERV-W processed pseudogenes harbor the *pre-gag* region, the *pre-gag* region might represent an intron and may have been the subject of alternative splicing at the time. The biological relevance of potential splicing within the *pre-gag* region and presence of a putative ORF within that region remains to be investigated.

Taken together, our analysis of the Catarrhini *pre-gag* region thus provided evidence for the evolutionary origin of about 60% of the sequence, specifically ~ 400 nt are shared with Platyrrhini ERV-W *pre-gag* and ~ 650 nt derive from the *pol* region of Platyrrhini HERVIP10-like sequences. When excluding an AG-rich region of about 140 nt (Fig. 5) greater than 30% of the Catarrhini ERV-W *pre-gag* sequence appears to have formed after the separation of Catarrhini and Platyrrhini, possibly through additional recombination events with a hitherto unidentified sequence partner (Fig. 4). BLAT searches of the human reference genome with the yet unexplained ~ 30% of the HERV-W *pre-gag* sequence as probe showed only very short (20–30 nt) stretches of sequence similarity with other repetitive elements (such as LINEs and MIR), yet subsequent RepeatMasker analysis did not corroborate the unexplained sequence portions as being derived from such repetitive elements (data not shown).

### Searching for a pre-gag region in other gammaretroviral HERV groups

Besides the HERV-W group [14], the presence of a *pre-gag* portion was previously reported for HERV-H gammaretroviruses [31]. Particularly, Jern and coauthors observed an unusually long 5′ leader sequence that precedes the traditional *gag* gene and includes an ORF positioned like the N terminus of murine leukemia virus (MLV) “glyco-Gag,” potentially encoding a proline and serine-rich domain remotely similar to MLV pp12 [31]. More in general, it is known that exogenous gammaretrovirus harbor a long 5′ leader region between the PBS and the start codon of the *gag* gene, and this element regulates central steps of viral replication, including splicing and - in some instances – ribosome occupancy [39].

**Fig. 6:**
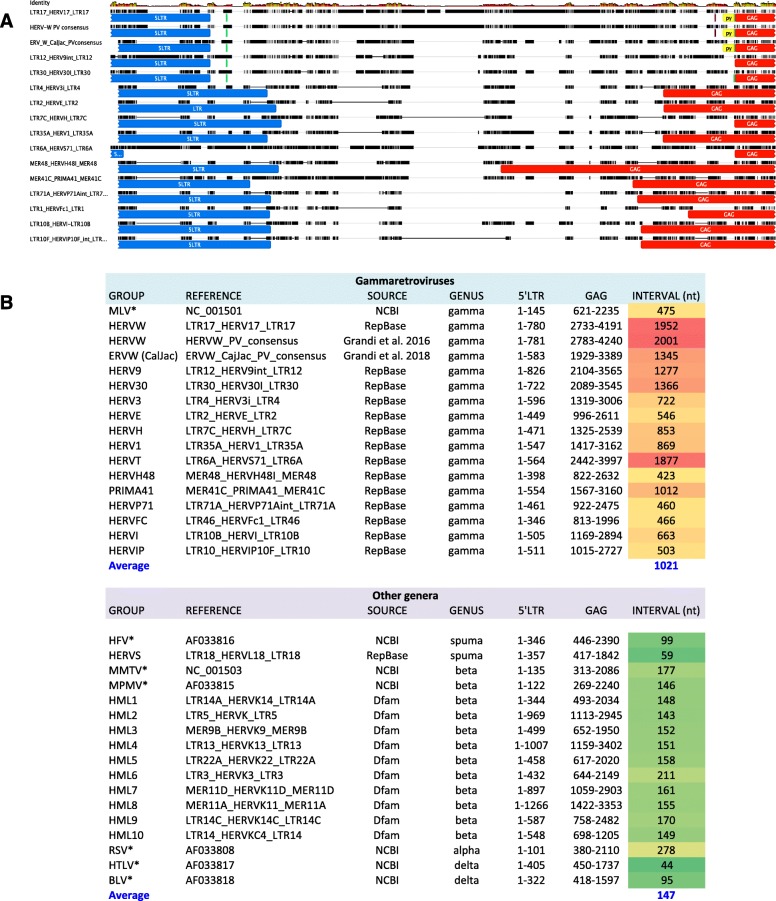
*Comparative analysis of pre-gag portions of gammaretroviruses.* Panel A: multiple alignment of the nucleotide sequence between 5’LTR (blue annotations) and *gag* gene (red annotations) of gammaretroviral HERV groups. Proviral reference sequences were retrieved from different sources, as indicated in the table below. Other annotations are referred to the identified intron: splice donor and acceptor sites (green), pyrimidine rich region (yellow), branch site (violet). Panel B: table reporting the characteristics of the sequences analyzed, including reference name/accession number, source, genus of belonging, nucleotide positions for 5’LTR and *gag* genes and length of the region between them (in nucleotides). Exogenous species are marked with an asterisk

Hence, we asked whether such a *pre-gag* region could be a common feature of all gammaretroviral HERVs, possibly suggesting a functional role of *pre-gag* also in the ancestral exogenous viruses. Proviral consensus sequences generated during characterization of the ERV-W group in the human genome [14] and marmoset genome [15] reference sequences were aligned with RepBase reference sequences of various human endogenous gammaretroviruses (HERV-W, HERV9, HERV30, HERV-H, HERV1, HERV3, HERV-E, HERV-T, HERV-H48, PRIMA41, HERVP71A, HERV-Fc1, HERVIP10F). As depicted in Fig. 6 (panel A), the *pre-gag* portion shared between Catarrhini and Platyrrhini ERV-W sequences showed partial nucleotide identity also in HERV9 and HERV30, possibly due to their closer sequence relationships with the ERV-W group. Of note, all the gammaretroviral HERV sequences taken into account showed an additional, intergenic portion between 5’LTR and *gag* gene, similarly to the ones already reported for HERV-H [31] and HERV-W [14] (Fig. 6). Such gammaretroviral HERV *pre-gag* region varied from 423 to about 2000 nucleotides in length, with an average value of 1021 bases. In contrast, the portion between 5’LTR and *gag* gene in the reference sequences of members of spumaretroviruses (including HERV-S) and betaretroviruses (including HERV-K HML1 to 10) as well as exogenous members of the HERV-devoid retroviral genera alpha- and deltaretroviruses was overall remarkably shorter, being only 147 nucleotides in average (Fig. 6, panel B). This further suggests that, even if showing divergent nucleotide sequence, the *pre-gag* portion is a stable feature of almost all gammaretroviral HERV groups, confirming a possible role in the latter biology that deserves dedicated investigation. At this regard, conserved SD and SA sites were identified also at the 5′ and 3′ ends (respectively) of the HERV-9 and HERV-30 *pre-gag* reference sequences (data not shown).

## Discussion

BLAT searches in marmoset and squirrel monkey Platyrrhini genome assemblies with the HERV-W group RepBase reference sequence (LTR17-HERV17-LTR17) as a query identified ERV sequences not previously considered in the ERV-W context. Respective sequences were already annotated as “ERV1–1_CJa-I” for the internal portion and “ERV1–1_CJa-LTR” for LTR sequences by Repeatmasker/RepBase, yet those sequences and the corresponding ERV group were not characterized in more detail so far, to the best of our knowledge.

Given that there is currently no taxonomical support and no correlation with other ERV1–1 groups annotated in RepBase for other vertebrates, and because of the high sequence identity with Catarrhini ERV-W elements and their close phylogenetic relationship at the amino acid level; we propose that the here characterized ERV sequences are members of the ERV-W group that colonized Platyrrhini species.

We have retrieved a total of 130 reasonably intact ERV loci with LTRs and flanking sequences from marmoset and squirrel monkey genome sequences and characterized these elements in terms of structure, phylogeny and estimated time of integration. Platyrrhini ERV-W sequences showed typical gammaretroviral structural features that they have in common with features already characterized in Catarrhini ERV-W sequences [14]. In particular, we identified i) an established Gag NC Zinc finger motif, ii) a second Gag NC Zinc finger motif presenting a modified amino acid sequence, as already reported for HERV-W [14] and HERV-H [31] elements, and iii) a GPY-F motif in Pol IN. These structural features are helpful with regard to phylogenetic relationships of retroviral sequences [27] and their presence in the here described Platyrrhini ERV sequences further support a close evolutionary relationship with the Catarrhini ERV-W group. We note that the great majority of Platyrrhini ERV-W elements harbor a PBS sequence predicted to bind tRNA^Arg^, as also observed for the HERV-W group [14]. Even if the canonical PBS type should be W one, such discrepancy was rather expected. In fact, the W PBS differs only slightly from R PBS, being found in a consistent number of HERV-9 and HERV-W elements, and sometimes the two codons may overlap due to a single nucleotide shift in the PBS sequence [4]. Accordingly, it has been shown that the PBS type has a weak taxonomic significance, with various major HERV groups (including HERV-H and HERV-L) presenting alternative PBS sequences [4].

In addition, Platyrrhini ERV-W loci are characterized by a *pre-gag* region that was previously reported to be present in almost all Catarrhini ERV-W sequences examined [14, 15]. Further sequence comparisons of Platyrrhini and Catarrhini *pre-gag* sequences now revealed high sequence similarities along the first 400 nucleotides, while Catarrhini ERV-W *pre-gag*, but not Platyrrhini ERV-W *pre-gag*, harbors a portion highly similar in sequence to a region within HERVIP10 *pol*. Of note, some ERV-W loci previously characterized in Catarrhini species’ Y chromosome [15] showed a *pre-gag* portion more similar to Platyrrhini *pre-gag*. It is conceivable that recombination events occurred early after the evolutionary split of the two parvorders, and more ancestral ERV-W sequences could likely be present in Y chromosome due to the fact that much of it does not recombine, except for intrachromosomal/inverted repeat-mediated recombination. Such low recombination rate has been already involved in the Y chromosome delayed loss of Alu transposons as compared with the autosomes, in which genomic redistributions of retroelements is greatly facilitated [40]. The presence of a long 5′ leader sequence between the PBS and *gag* has been reported to be an unique genetic feature of exogenous gammaretroviruses, providing splicing signals and promoting ribosome synthesis of viral proteins independently of the 5′ cap structure through an internal ribosome entry site (IRES) [39]. A similar 5′ leader region has been identified also in some class I mammalian ERV groups, including anthropoids HERV-T, HERV-I, and HERV-3 [39] as well as HERV-H [31]. We extended the analysis including a total of 14 gammaretroviral HERV groups, which have been compared with class II and III HERV sequences. Intriguingly, the presence of a long intergenic region (from 423 to about 2000 nucleotides) between 5’LTR and *gag* has been found in all the gammaretroviral HERV groups analyzed, being instead absent in the other genera. This stable feature shared by ancient and existing gammaretroviruses further corroborates an important role in their replication cycle. Accordingly, MLV, feline leukemia virus, and koala retrovirus all harbor additional ORFs that are translated in the 5′ leader and encode a glycosylated form of Gag, enhancing the infectivity of the viruses [39]. Similarly, HERV-H *pre-gag* includes an ORF positioned like the N terminus of MLV *gag*, possibly encoding for a MLV pp12-like protein [31]. Our analysis furthermore identified a putative intron within the ERV-W *pre-gag* portion in both Catarrhini and Platyrrhini species, yet located in different subregions within *pre-gag* and thus showing a different nucleotide sequence. Identification of a small subset of Catarrhini ERV-W processed pseudogenes lacking the *pre-gag* region and presence of putative splicing donor and acceptor sites at the *pre-gag* 5′ and 3′ ends, respectively, suggests an alternative splicing strategy for the ancestral retroviral sequences. Overall, the fact that the ERV-W *pre-gag* harbors a putative ORF, presenting also splicing signals that occasionally led to the removal of such portion in ERV-W-derived processed pseudogenes, could indicate a similar function originally crucial for viral replication, and possibly removed by intronic splicing after endogenization due to the loss of replication competence in favor of a more compact (and hence transposable) genetic structure. Such strategy was already observed regarding the frequent loss of the *env* gene, a trait that together with retrotransposition led ERVs to became genomic superspreaders [41]. Further studies are needed to assess the biological relevance of the *pre-gag* region and splicing within that region in ERV-W and other gammaretroviral ERVs.

Platyrrhini ERV-W sequences were furthermore different from Catarrhini ERV-W in that there was no evidence of ERV-W loci being processed pseudogenes, that is ERV-W loci having been generated by LINE-1-mediated retrotransposition, which accounted indeed for approximately two-thirds of HERV-W loci in the human genome [14, 19, 24]. Absence of processed pseudogenes for Platyrrhini ERV-W was rather unexpected considering overall high nucleotide identities with Catarrhini ERV-W, and because LINE-1-mediated formation of ERV-W processed pseudogenes in other primate lineages appeared to have been ongoing for an extended period of time [15]. One might speculate that some minor, so far unidentified nucleotide differences in ERV-W proviral sequences and transcripts prevented retrotransposition by LINE-1 machinery, or there was insufficient LINE-1 activity in respective species evolutionary lineages at the time. Accordingly, LINE-1 activity was recently reported to be low among Atelidae, and large differences in LINE-1 activity were noted for various Platyrrhini lineages [42]. However, it currently appears difficult to conclude that such variable, potentially low LINE-1 activity indeed caused the observed lack of formation of ERV-W processed pseudogenes during the evolution of Platyrrhini. Additional analyses will be required to explain the lack of formation of ERV-W processed pseudogenes in the Platyrrhini lineage.

Phylogenetic analysis of marmoset and squirrel monkey ERV-W LTRs revealed at least 2 LTR subgroups, named A and B, that support the evolution of different LTRs associated with monophyletic proviral bodies, as already reported for Catarrhini ERV-W proviruses [14, 15]. In the same line, Catarrhini and Platyrrhini ERV-W elements are highly identical in sequence for the internal proviral portions, and rather divergent in sequence with regard to LTR sequences and the *pre-gag* region.

The time period of integration of Platyrrhini ERV-W sequences into host genomes was estimated to have taken place between 25 and 15 mya, with the earlier provirus formations being associated with LTRs of subgroup A followed by the major wave of provirus formations with LTRs of subgroup B. The time period of genome colonization was furthermore supported by presence of orthologous ERV-W-like loci shared between marmoset and squirrel monkey genomes as well as related ERV-W elements in other Platyrrhini species belonging to Cebidae and Atelidae lineages.

## Conclusions

Besides Catarrhini species, Platyrrhini primates belonging to both Cebidae and Atelidae families were colonized by ERV-W as well, approximately between 25 and 15 mya. Such colonization has been sustained by at least two different ERV-W subgroups, which can be distinguished by alternative LTR types that were furthermore different in sequence from Catarrhini ERV-W LTRs, indicating that various ERV-W versions have colonized respective primate lineages. The *pre-gag* region and an intron located within *pre-gag* appears as a common feature of the ERV-W group, and the biological relevance of this proviral region deserves further investigation especially with regard to the biology of ancestral gammaretroviruses.

## Methods

### ERV sequences and primate species included in the analyses

ERV-W like elements analyzed in this study were retrieved as previously described [15]. Briefly, a number of ERV-W-like elements were retrieved from UCSC Genome Browser [21] after identification by BLAT searches [22] using as a query the HERV-W group reference sequences (HERV17 and LTR17) from RepBase Update [23] for the following Platyrrhini genome assemblies: marmoset (*Callithrix jacchus*, assembly March 2009 - WUGSC 3.2/calJac3) and squirrel monkey (*Saimiri boliviensis*, assembly Oct. 2011 - Broad/saiBol1). Sequences identified by BLAT searches have been annotated in the UCSC Genome Browser by RepeatMasker/RepBase [23] as ERV1–1_CJa-I for the internal portion and ERV1–1_CJa-LTR for the LTRs. ERV-W-like sequences were retrieved including 500 nucleotides of 5′ and 3′-flanking sequence portions each. A total of 130 proviral sequences harboring relatively intact retroviral genes and LTRs, based on pairwise dot-plot comparisons with a proviral reference built assembling the above ERV1–1_CJa-I with flanking ERV1–1_CJa-LTRs, were selected for subsequent analysis [15]. We also estimated the number of solitary LTRs by BLAT searching each primate genome assembly with an LTR reference sequence as probe. We retrieved matching sequences including 500 nt of 5′ and 3′ flanking sequence portions each. Retrieved sequences were then multiply aligned together with reference sequences consisting of the 5′-most 1000 nt and the 3′-most 1000 nt of the full-length proviral consensus sequence. This allowed us to distinguish proviral LTRs from putative solitary LTRs based on presence or absence, respectively, of internal proviral regions. To further verify results, we also intersected and compared the genomic coordinates of BLAT matches from LTR searches with those of the analyzed proviruses, likewise identifying coordinates representing proviral loci and solitary LTRs. Similarly, to assess the presence of orthologous ERV-W-like loci shared by marmoset and squirrel monkey, we have downloaded the 59 and 71 proviral loci taken into account, respectively, adding to each nucleotide sequence 500 bp flankings at 5′ and 3′ ends. Then we have performed multiple alignments to check the presence of shared flanking sequences, suggesting that the two loci can be orthologs. To check our results, we also made comparative genomic analyses with Genome Browser “Lift Over” tool, identifying for each ERV-W-like locus in marmoset and squirrel monkey the corresponding genomic positions in the human reference genome sequence (GRCh38/hg38) and by comparing the obtained coordinates (we did not make direct comparison between marmoset and squirrel monkey ERV-W-like loci coordinates because these assemblies are not included in Genome Browser comparative genomics tools).

Besides the above marmoset and squirrel monkey reference genome assemblies, presence of ERV-W like elements was also assessed in other Platyrrhini species belonging to Cebidae, Atelidae and Pitheciidae lineages by Blast searches of nucleotide collection (nt) database of the National Center for Biotechnogy Information (NCBI), using discontiguous megablast and a sequence comprised of ERV1–1 CJa-LTR–CJa-I–CJa-LTR as query.

### Pairwise and multiple sequence alignments

Nucleotide sequences were pairwisely and multiply aligned using Geneious bioinformatics software, version 8.1.4 [43] applying MAFFT algorithms FFT-NS-i × 1000 or G-INS-I [44] with default parameters. Generated alignments were visually inspected and, when necessary, manually optimized before subsequent analyses. For pairwise alignments, the dot-plot analysis tool implemented in Geneious was used for visual comparisons of sequences. Graphical representations of alignments were generated with Geneious bioinformatics software and adapted as appropriate.

### Structural characterization of ERV sequences

Compiled ERV-W-like sequences were multiply aligned and compared to an LTR17-HERV17-LTR17 proviral reference, obtained from RepBase Update [23]. All the ERV-W-like elements were analyzed for the presence of conserved features with taxonomic significance, i.e. i) the nucleotide sequence of the primer binding site (PBS), ii) the Gag nucleocapsid (NC) zinc finger amino acid motif, iii) the Pol Integrase (IN) C-terminal GPY/F amino acid motif and iv) any bias in the overall nucleotide composition along the sequence [27]. The PBS assignment to the corresponding tRNA type was by similarity analysis employing a tRNA sequence library built from the Transfer RNA database (tRNAdb) hosted at Leipzig University [45] and from a PBS sequence library generated in our previous classification study [4].

### Phylogenetic analyses

Phylogenetic analyses were performed from manually optimized sequence alignments using MEGA Software, version 6 [46]. Phylogenetic trees were inferred using either Neighbor Joining (NJ) and/or Maximum Likelihood (ML) statistical methods. NJ trees were built using p-distance or Kimura 2-parameter models applying pairwise deletion, and phylogenies were further assessed by the bootstrap method with 1000 replicates. ML trees were built using a Poisson correction model, and phylogeny was tested by the bootstrap method with 1000 replicates.

### Time of integration estimation

The time of integration of each ERV sequence was estimated through different approaches, all based on the percentage of divergent nucleotides (D) as calculated by MEGA software (version 6) [46]. D was estimated after removal of hypermutating CpG dinucleotides, using a p-distance model and applying pairwise deletion for the following categories of pairwisely aligned sequences: i) the 5′ and 3’LTR of each provirus, ii) proviral *gag* gene and a consensus generated for each subgroup (only for sequences that were included in subgroup A and B based on LTR phylogeny). The obtained D values were employed following previous methodologies [47] to estimate time of integration (T) of each ERV1–1 sequence, based on the equation

$$ \mathrm{T}=\mathrm{D}/\mathrm{SR} $$

where SR corresponds to the estimated neutral substitution rate acting on the host genome (0.0045 substitutions/nucleotide/million years) [36].

T values obtained from 5′ and 3’LTR D calculations were divided by a factor of 2, considering that each LTR evolved independently in the genome (T = D/SR/2). The resulting age of each sequence was expressed as the average of T obtained from the different approaches, excluding values with a standard deviation > 20%.

### Inference of the putative gammaretroviral RT-RH amino acid sequence

Putative Reverse Transcriptase - Ribonuclease H (RT-RH) amino acid sequences of retrieved ERV-W proviruses and the other gammaretroviral ERV groups were inferred as follows. RT-RH portions in the HERV-W sequences and in the Platyrrhini ERV-W-like elements were identified in the respective proviral consensus sequences [14, 15] using multiple approaches that included i) RetroTector online ORF prediction and conserved domain identification (http://retrotector.neuro.uu.se/) [26]; ii) NCBI Conserved Domain search tool (https://www.ncbi.nlm.nih.gov/Structure/cdd/wrpsb.cgi) [38]; and iii) *pol* ORF sequence translation and comparison with Pol protein sequences of other gammaretrovirus-like HERVs, as reconstructed from the most intact insertions present in human genome assembly GRCh37/hg19 by RetroTector software [4].

## Supplementary information

**Additional file 1: Figure S1.** Graphical representation of the phylogeny of primates. Primate species addressed in the present study are indicated, including chimpanzee (*Pan troglodytes*), gorilla (*Gorilla gorilla gorilla*), orangutan (*Pongo pygmaeus abelii*), gibbon (*Nomascus Leucogenys*), various old world monkeys (OWM), marmoset (*Callithrix jacchus*), and squirrel monkey (*Saimiri boliviensis*). Numbers near nodes represent evolutionary divergence times of lineages (in millions of years ago) as estimated previously [16, 17].

**Additional file 2: Figure S2.** Phylogenetic analysis of Marmoset and Squirrel Monkey ERV-W LTR sequences. Nucleotide sequences of Platyrrhini proviral 5′ and 3′ LTRs of marmoset and squirrel monkey ERV-W elements were multiply aligned and analyzed using the Neighbor-joining method and the Kimura-2-parameter model the applying pairwise deletion option. Phylogeny was tested using the Bootstrap method with 1000 replicates. The length of branches indicates the number of substitutions per site. LTR subgroups (see the main paper text) are indicated by squared brackets.

**Additional file 3: Figure S3.** Phylogenetic analysis of the RT-RH region. Platyrrhini and Catarrhini ERV-W RT-RH amino acid sequences (black and white triangles, respectively) were inferred and translated bioinformatically from respective proviral consensus sequences, as detailed in materials and methods. RT-RH sequences of other gammaretroviral-like HERVs derive from amino acid consensus sequences reconstructed previously by RetroTector software [4]. RT-RH amino acid sequences were analyzed using the Maximum likelihood method and Poisson model. Phylogeny was tested using the Bootstrap method with 1000 replicates. Length of branches indicates the number of substitutions per site.

**Additional file 4: Figure S4.** Splice signals in the pre-gag region. Panel A: 5′ and 3′ ends of HERV-W *pre-gag* regions display striking similarities with sequences of splice donor (SD) and splice acceptor (SA) sites. A multiple sequence alignment of HERV-W loci lacking the *pre-gag* region and HERV-W consensus sequences harboring the *pre-gag* region is shown. Note that only relevant 5′ and 3′ parts of the *pre-gag* region are depicted. Sequence logos depicting sequence conservation of SD and SA sites are shown. Note the similarities with sequences included in the multiple sequence alignment, supporting the idea that the *pre-gag* 5′ and 3′ ends represent intron ends. Panel B: further comparison of conserved splice signal sequences with HERV-W sequences identified in [14]. SD = splice donor site, B = branch site, SA = splice acceptor site, pu = purine, py = pyrimidine. Sequence logos indicate the frequency of each particular nucleotide among proviral sequences. Numbers at the bottom indicate the number of proviral sequences, among the ones with the *pre-gag* region, having the particular nucleotide.

**Additional file 5.** Fasta alignment of ERV-W proviral sequences retrieved from marmoset and squirrel monkey genome assemblies. A total of 59 and 71 reasonably complete ERV-W proviruses, i.e. having intact LTRs and internal portions, were retrieved from marmoset and squirrel monkey genome assemblies, respectively, and aligned with respect to the corresponding proviral consensus sequences [15].

## Data Availability

All data generated or analyzed during this study are either publically available or included in this published article and its supplementary information files.
