# Supplementary figures and images for: Identification and characterization of ERV-W-like sequences in Platyrrhini species provides new insights into the evolutionary history of ERV-W in primates
Source: Mob DNA. 2020 Feb 1;11:6. doi: 10.1186/s13100-020-0203-2 (PMC6995185; doi:10.1186/s13100-020-0203-2)

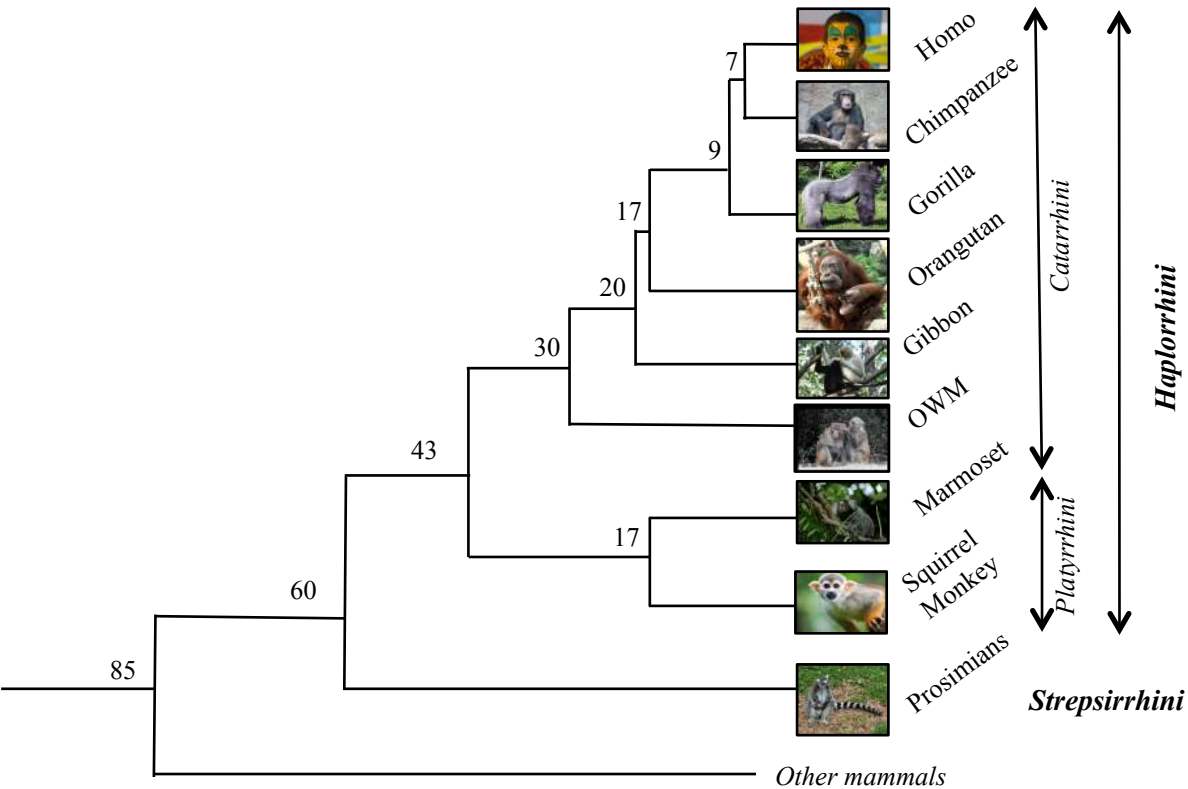

Supplement: Supplementary file 1 — Additional file 1: Figure S1. Graphical representation of the phylogeny of primates. Primate species addressed in the present study are indicated, including chimpanzee (Pan troglodytes), gorilla (Gorilla gorilla gorilla), orangutan (Pongo pygmaeus abelii), gibbon (Nomascus Leucogenys), various old world monkeys (OWM), marmoset (Callithrix jacchus), and squirrel monkey (Saimiri boliviensis). Numbers near nodes represent evolutionary divergence times of lineages (in millions of years ago) as estimated previously [16, 17]. [file 13100_2020_203_MOESM1_ESM.pdf]

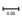

Supplement: Supplementary file 2 — Additional file 2: Figure S2. Phylogenetic analysis of Marmoset and Squirrel Monkey ERV-W LTR sequences. Nucleotide sequences of Platyrrhini proviral 5′ and 3′ LTRs of marmoset and squirrel monkey ERV-W elements were multiply aligned and analyzed using the Neighbor-joining method and the Kimura-2-parameter model the applying pairwise deletion option. Phylogeny was tested using the Bootstrap method with 1000 replicates. The length of branches indicates the number of substitutions per site. LTR subgroups (see the main paper text) are indicated by squared brackets. [file 13100_2020_203_MOESM2_ESM.pdf]

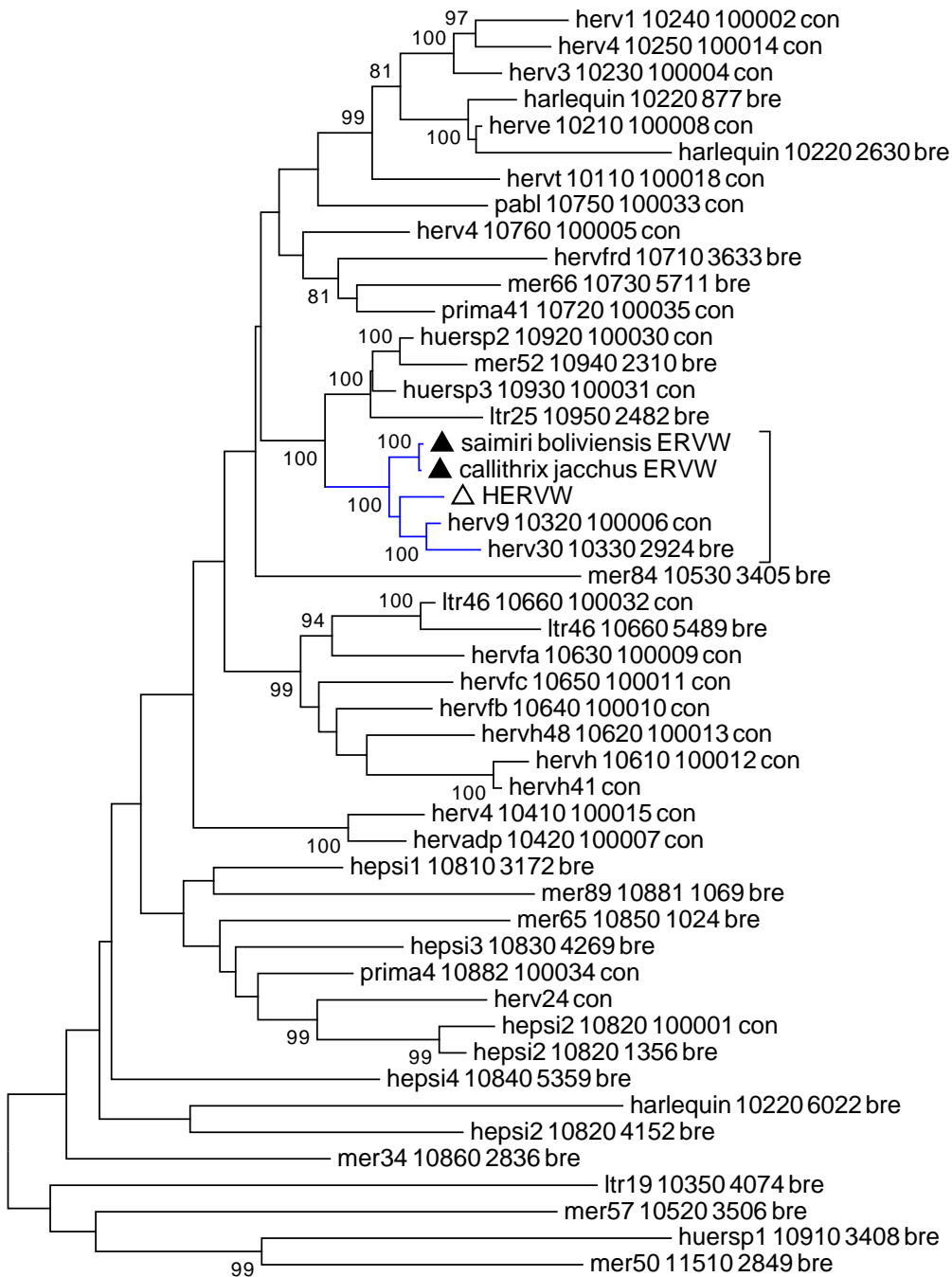

0.5

Supplement: Supplementary file 3 — Additional file 3: Figure S3. Phylogenetic analysis of the RT-RH region. Platyrrhini and Catarrhini ERV-W RT-RH amino acid sequences (black and white triangles, respectively) were inferred and translated bioinformatically from respective proviral consensus sequences, as detailed in materials and methods. RT-RH sequences of other gammaretroviral-like HERVs derive from amino acid consensus sequences reconstructed previously by RetroTector software [4]. RT-RH amino acid sequences were analyzed using the Maximum likelihood method and Poisson model. Phylogeny was tested using the Bootstrap method with 1000 replicates. Length of branches indicates the number of substitutions per site. [file 13100_2020_203_MOESM3_ESM.pdf]

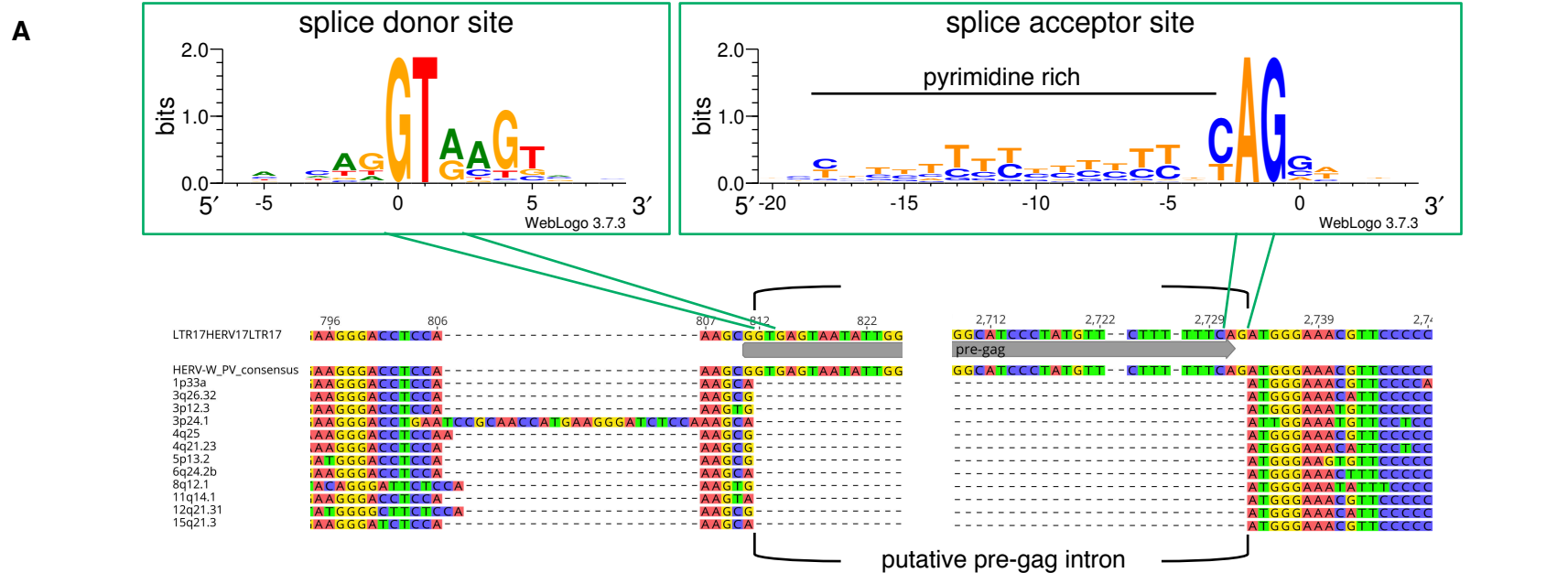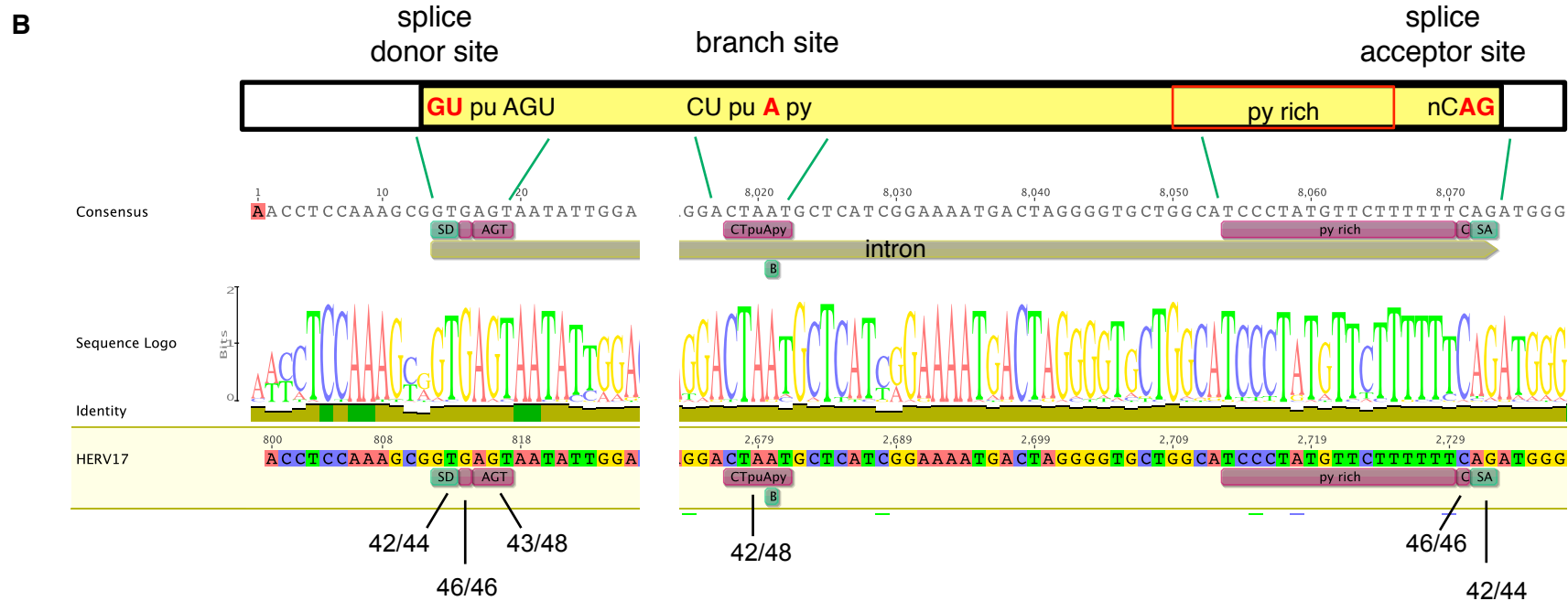

Supplement: Supplementary file 4 — Additional file 4: Figure S4. Splice signals in the pre-gag region. Panel A: 5′ and 3′ ends of HERV-W pre-gag regions display striking similarities with sequences of splice donor (SD) and splice acceptor (SA) sites. A multiple sequence alignment of HERV-W loci lacking the pre-gag region and HERV-W consensus sequences harboring the pre-gag region is shown. Note that only relevant 5′ and 3′ parts of the pre-gag region are depicted. Sequence logos depicting sequence conservation of SD and SA sites are shown. Note the similarities with sequences included in the multiple sequence alignment, supporting the idea that the pre-gag 5′ and 3′ ends represent intron ends. Panel B: further comparison of conserved splice signal sequences with HERV-W sequences identified in [14]. SD = splice donor site, B = branch site, SA = splice acceptor site, pu = purine, py = pyrimidine. Sequence logos indicate the frequency of each particular nucleotide among proviral sequences. Numbers at the bottom indicate the number of proviral sequences, among the ones with the pre-gag region, having the particular nucleotide. [file 13100_2020_203_MOESM4_ESM.pdf]
